# Correction: Salakphet et al. Molecular Classification and Clinical Outcomes in Endometrial Cancer: Real-World Evidence from a Tertiary Care Center. *Cancers* 2026, *18*, 181

**DOI:** 10.3390/cancers18111785

**Published:** 2026-05-29

**Authors:** Tanadon Salakphet, Prapaporn Suprasert, Tip Pongsuvareeyakul, Chinachote Teerapakpinyo, Surapan Khunamornpong

**Affiliations:** 1 Division of Gynecologic Oncology, Department of Obstetrics and Gynecology, Faculty of Medicine, Chiang Mai University, Chiang Mai 50200, Thailand; tanadon.s@cmu.ac.th; 2 Division of Gynecologic Pathology, Department of Pathology, Faculty of Medicine, Chiang Mai University, Chiang Mai 50200, Thailand; tang_tip@hotmail.com (T.P.); skhunamo@yahoo.com (S.K.); 3 Chula GenePRO Center, Faculty of Medicine, Chulalongkorn University, Bangkok 10330, Thailand; chinachote.t@chula.ac.th

**Error in Table**

In the original publication [1], there was a mistake in Table 2 as published. The final line of Table 2 was omitted. The corrected Table 2 appears below. The authors state that the scientific conclusions are unaffected. This correction was approved by the Academic Editor. The original publication has also been updated.

## Figures and Tables

**Table 2:** Univariate and Multivariate Logistic Regression Analysis of Clinicopathological Factors Associated with MMR and p53 Status.

Factors	MMR							p53						
	dMMR (%)	pMMR (%)	Total	Unadjusted OR * (95%CI)	*p* Value	Adjusted OR ** (95%CI)	*p* Value	Wild	Abnormal	Total	Unadjusted OR * (95%CI)	*p* Value	Adjusted OR ** (95%CI)	*p* Value
Age (years)														
≤60 years	52 (81.3)	12 (18.8)	64 (63.4)	3.683 (1.496–9.070)	0.006	2.611 (0.888–7.677)	0.081	33 (61.1)	21 (38.9)	54 (40.6)	5.731 (2.663–12.332)	<0.001	7.952 (1.208–47.697)	0.031
>60 years	20 (54.1)	17 (45.9)	37 (36.6)					17 (21.5)	62 (78.5)	79 (59.4)				
BMI														
<25	47 (75.8)	15 (24.2)	62 (61.4)	1.755 (0.731–4.210)	0.260	-	-	32 (43.8)	41 (56.2)	73 (54.9)	1.821 (0.886–3.742)	0.109	-	-
≥25	25 (64.1)	14 (35.9)	39 (38.6)					18 (30.0)	42 (70.0)	60 (45.1)				
UD														
None	28 (82.4)	6 (17.6)	34 (33.7)	2.439 (0.883–6.736)	0.104	-	-	25 (67.6)	12 (32.4)		5.917 (2.519–13.509)	<0.001	5.618 (0.870–36.278)	0.070
Present	44 (65.7)	23 (34.3)	67 (66.3)					25 (26.0)	71 (74.0)					
Residual disease														
None	65 (70.7)	27 (29.3)	92 (91.1)	0.688 (0.134–2.526)	0.727	-	-	45 (37.5)	75 (62.5)	120 (90.2)	0.960 (0.269–3.114)	1.000	-	-
Present	7 (77.8)	2 (22.2)	9 (8.9)					5 (38.5)	8 (61.5)	13 (9.8)				
Histology														
Endometrioid	65 (86.7)	10 (13.3)	75 (74.3)	17.463 (5.915–52.622)	<0.001	15.215 (4.992–46.374)	<0.001	46 (80.7)	11 (19.3)	57 (42.9)	75.273 (22.611–250.586	<0.001	79.416 (10.599–595.049)	<0.001
Non-endometrioid	7 (26.9)	19 (65.5)	26 (25.7)					4 (5.3)	72 (94.7)	76 (57.1)				
MI														
Less than 50%	39 (76.5)	12 (23.5)	51 (50.5)	1.674 (0.700–4.006)	0.277	-	-				1.266 (0.625–2.563)	0.590	-	-
≥50%	33 (66.0)	17 (28.7)	50 (49.5)					26 (35.1)	48 (64.9)	74 (55.6)				
LVSI														
None	45 (73.8)	16 (26.2)	61 (60.4)	1.354 (0.565–3.244)	0.509	-	-				0.627 (0.309–1.275)	0.210	-	-
Present	27 (67.5)	13 (32.5)	40 (39.6)					25 (50.0)	32 (56.1)	57 (42.9)				
Peritoneal cytology														
Negative	70 (72.9)	26 (27.1)	96 (95.0)	4.038 (0.638–25.555)	0.141	-	-	48 (40.7)	70 (59.3)	118 (88.7)	4.457 (0.962–20.653)	0.050	-	-
Positive	2 (40.0)	3 (60.0)	5 (5.0)					2 (13.3)	13 (86.7)	15 (11.3)				
Stage ***														
I&II	47 (78.3)	13 (21.7)	60 (59.4)	2.314 (0.962–5.568)	0.074	-	-	33 (44.0)	42 (56.0)	75 (56.4)	1.895 (0.917–3.918)	0.105	-	-
III&IV	25 (61.0)	16 (39.0)	41 (40.6)					17 (29.3)	41 (70.7)	58 (43.6)				
Risk														
Low&intermediate	44 (89.8)	5 (10.2)	49 (48.5)	7.543 (2.578–22.072)	<0.001	1.329 (0.298–5.919)	0.709	31 (81.6)	7 (18.4)	38 (28.6)	17.714 (6.769–46.357)	<0.001	1.848 (0.059–57.817)	0.727
High risk	28 (53.8)	24 (46.2)	52 (51.5)					19 (20.0)	76 (80.0)	95 (71.4)				
Adjuvant RT														
None	31 (77.5)	9 (22.5)	40 (75.5)	0.287 (0.03–2.516)	0.419	-	-	19 (32.2)	40 (67.8)	59 (84.3)	0.048 (0.006–0.398)	<0.001	0.268 (0.020–3.600)	0.320
Present	12 (92.3)	1 (7.7)	13 (24.5)					10 (90.9)	1 (9.1)	11 (15.7)				
Adjuvant CT														
None	66 (74.2)	23 (25.8)	89 (88.1)	2.870 (0.841–9.789)	0.098	-	-	44 (44.9)	54 (55.1)	98 (73.7)	3.938 (1.500–10.337)	0.004	0.546 (0.037–8.013)	0.659
Present	6 (50.0)	6 (50.0)	12 (11.9)					6 (17.1)	29 (82.9)	35 (26.3)				

* Chi-square or Fisher’s exact test. ** Binary regression (Backward, likelihood ratio). *** Figo stage 2018. MMR, mismatch repair gene; dMMR, deficient mismatch repair; pMMR, proficient mismatch repair; OR, odds ratio; CI, confidence interval; BMI, body mass index; UD, underlying disease; MI, myometrial invasion; LVSI, lymphovascular space invasion; RT, radiation therapy; CT, chemotherapy.
