# Supplementary material for: Molecular Classification and Clinical Outcomes in Endometrial Cancer: Real-World Evidence from a Tertiary Care Center
Source: Cancers (Basel). 2026 Jan 6;18(2):181. doi: 10.3390/cancers18020181 (PMC12838868; doi:10.3390/cancers18020181)
Supplement: Supplementary file 1 [file cancers-18-00181-s001.zip › cancers-4058929-supplementary.pdf]

Supplement table

Table S 1: Clinical characteristics (N=184)

	N(%)
Mean age (+SD): years	61.26(+9.70)
Mean body mass index (+SD): mg/kg	24.48(+5.08)
Clinical presenting	
Abnormal uterine bleeding	30(16.3)
Postmenopausal bleeding	152(82.6)
Pelvic mass	2(1.1)
Underlying disease	
None	50(27.2)
DM+HT+DLP	24(13.0)
HT	21(11.4)
HT+DLP	18(9.8)
DM+HT	10(5.4)
Other *	61(33.2)
Coexisting cancer	
None	156(84.8)
Breast cancer	12(6.5)
Colon cancer	7(3.8)
Cervical cancer	2(1.1)
Ovarian cancer	2(1.1)
Rectal caner	2(1.1)
Lung cancer	1(0.5)
Hepatocellular carcinoma	1(0.5)
Breast cancer + colon cancer	1(0.5)
Fractional curettage/Endometrial sampling	
Not done	13(7.1)
Done	171(92.9)
Type of surgery	
Laparotomy	165(89.7)
Laparoscopy	19(10.3)
Pelvic node sampling	138(75.0)
Paraaortic node sampling	48(26.6)
Residual disease	
No residual disease	169(91.8)
Residual disease	15(8.2)
Figo Stage (2018)	
IA	60(32.6)
IB	47(25.5)
II	11(6.0)
IIIA	16(8.7)
IIIB	7(3.8)
IIIC1	16(8.7)
IIIC2	10(5.4)
IVB	17(9.2)
Final histology	
Endometrioid	105(57.1)

High grade serous cancer<br>Carcinosarcoma<br>Clear cell CA	56(30.4)<br>11(6.0)<br>12(6.5)
Tumor grade<br>Grade 1<br>Grade 2<br>Grade 3	 43(23.4)<br>25(13.6)<br>116(63.0)
Myometrial invasion<br>Endometrium<br>Less than 50%<br>Equal/more than 50%<br>Sub-serosa	 7(3.8)<br>71(38.6)<br>71(38.6)<br>35(19.0)
LVSI<br>None<br>Focal<br>Substantial	 75(40.8)<br>40(21.7)<br>69(37.5)
Median LVSI	7.00(1-100)
Endocervical involvement	43(23.4)
Pelvic node involvement	27(14.7)
Paraaortic node involvement	7(3.8)
Peritoneal washing positive	15(8.2)
Risk based<br>Low risk<br>Intermediate risk<br>High intermediate risk<br>High risk	 20(10.9)<br>29(15.8)<br>29(15.8)<br>106(57.6)
Neoadjuvant chemotherapy	14(7.6)
Adjuvant treatment<br>None<br>Yes	 38(20.7)<br>146(79.3)
Adjuvant radiation<br>None<br>VBT<br>EBRT<br>BBRT + VBT<br>Sequential chemotherapy -> EBRT<br>Chemotherapy -> EBRT -> Chemotherapy	 73(39.7)<br>25(13.6)<br>6(3.3)<br>26(14.1)<br>52(28.3)<br>2(1.1)
Adjuvant chemotherapy<br>None<br>Carboplatin + paclitaxel<br>Carbolpatin<br>Adriamycin + ifosfamide<br>Adriamycin	 94(51.1)<br>83(45.1)<br>3(1.6)<br>1(0.6)<br>1(0.6)
Outcome<br>Recurrence<br>Death	 29(15.8)<br>52(28.3)

*other: Diabetes mellitus (DM)(2), dyslipidemia(DLP)(2) ,hyperthyroidism(1), hypothyroidism(3), heart disease(4),CA breast(8),CAlung(1),obstructive sleep apnea(OSA)(1),end stage renal disease (ESRD)(2), asthma(2),CA colon (5),hepato- cellular cancer (1), CA cervix (2),hyperthyroid+CA ovary (1),hypertension(HT)+DLP+coronary artery disease (CAD)(3), CA ovary(1),HT+DLP+chronic

kidney disease (CKD)+CAD (1), HT+DLP+old cerebrovascular accident (CVA)(1), HT+CA colon(2), valvular heart disease (VHD)+ hypothyroidism (1),DM+ asthma (1), DM+DLP(1), HT+VHD(2), DM+HT+ESRD(2),HT+gout(1), DM+HT+DLP+abdominal aortic aneurysm (1), DM+HT+CA breast + CA colon (1), DM+HT+DLP+old CVA+CA rectum(1), HT+CA breast (4), DM+HT+triple vessel disease+ old CVA (1),HT+DLP+CA rectum(1)

DM,diabetes mellitus;**HT**,hypertension;DLP, dyslipidemia; **LVSI**, lymphovascular space invasion; **VBT**, vaginal brachytherapy; **EBRT**, external beam radiation therapy

Table S2: The clinicopathologic characteristics of no specific molecular profile (NSMP) endometrial cancer patients

SN	Age (years)	BMI (kg/m2)	Underlying disease	Operation	Histology (grade)	MI	LVSI (foci)	Stage	Risk	Adjuvant treatment	Recurrence	PFS (years)	OS (years)
1	55	33.8	HT	TLH&BSO (uterine morcellation)	EM(2)	<50%	none	IA	high	PT x 6 → EBRT+VBT	No	1.77	2.09
2	60	20.4	HT	TAH&BSO&PLS	EM(1)	>50%	Focal (7)	IB	HIR	PTx6→ EBRT+VBT	No	2.02	2.23
3	61	24.3	No	TLH&BSO^PLS&PANS	EM(1)	>50%	Focal (5)	IB	HIR	EBRT+VBT	No	1.79	2.14

SN, serial number; **BMI**, body mass index; **MI**, myometrial invasion; **LVSI**, lymphovascular space invasion; **PFS**, progression free survival; **OS**, overall survival; **HT**, hypertension; **TLH**, total laparoscopic hysterectomy; **BSO**, bilateral salpingo-oophorectomy; **EM**, endometrioid cancer; **HIR**, high intermediate risk; **PT**, carboplatin + paclitaxel ; **EBRT**, external beam radiation therapy; **VBT**, vaginal brachytherapy

Table S3: Summary of Clinical, Pathological, and Molecular Data of DNA polymerase epsilon (POLE)-Mutated Cases

SN	Age (years)	BMI	UD	Histology (grade)	MI	LVSI (Number)	Stage (risk)	Other test	Idella (genotype)	Bi-directional Sanger sequencing (genotype)	Adjuvant treatment	Outcome	PFS(years)	OS(years)
155	69	28.19	HT+DLP	EM (1)	≥50%	No	IB (IR)	Not done	Detect Exon 9: P286H/P286L/P286S	Not detect	VBT	NED	3.12	3.54
156	75	30.83	HT	EM (1)	≥50%	No	IB (IR)	Not done	Detect Exon 9: P286R/M295R/S297F	Detect Exon 9 c.857C>G p.(Pro286Arg)	VBT	NED	0.50	3.37
157	57	22.44	No	EM (3)	≥50%	Focal (1 space)	IB (HIR)	dMMR,wild type p53	Invalid	Detect Exon 14 c.1397C>T p.(Thr466Ile)	EBRT+VBT	NED	2.72	3.22
158	67	24.73	No	EM (1)	≥50%	Substantial (20 spaces)	IB (IR)	Not done	Invalid	Invalid	VBT	NED	2.82	3.05
159	80	21.33	HT+DLP	EM (2)	≥50%	Focal (2 spaces)	IB (IR)	Not done	Detect Exon 9: P286R/M295R/S297F	Detect Exon 9 c.857C>G p.(Pro286Arg)	VBT	NED	2.73	2.85
160	56	18.55	No	EM (3)	<50%	No	IA (IR)	Wild type p53	Detect Exon 9: P286R/M295R/S297F	Detect Exon 9 c.857C>G p.(Pro286Arg)	VBT	NED	2.61	2.72
161	56	27.91	No	EM (1)	≥50%	Substantial (9 spaces)	IB (HIR)	Not done	Detect Exon 9: P286H/P286L/P286S	Not detect	EBRT+VBT	NED	2.56	2.64
162	62	25.68	HT+DLP	EM	<50%	No	IB	Not done	Detect	Not detect	EBRT+VBT	NED	2.41	2.92

				(3)			(HIR)		Exon 9: P286H/P286L/P286S					

BMI, body mass index ; **UD,** underlying disease; MI, myometrial invasion; **LVSI**, lymphovascular space invasion;**PFS**, progression free survival; **OS**, overall survival;**EM**, endometrioid cancer;**dMMR**, deficient mismatch repair;**EBRT**, external beam radiation therapy; **VBT**, vaginal brachytherapy
